# MicroRNA-155 Deficiency Attenuates Liver Steatosis and Fibrosis without Reducing Inflammation in a Mouse Model of Steatohepatitis

**DOI:** 10.1371/journal.pone.0129251

**Published:** 2015-06-04

**Authors:** Timea Csak, Shashi Bala, Dora Lippai, Karen Kodys, Donna Catalano, Arvin Iracheta-Vellve, Gyongyi Szabo

**Affiliations:** 1 Department of Medicine, University of Massachusetts Medical School, Worcester, Massachusetts, United States of America; 2 2nd Department of Medicine, Semmelweis University, Budapest, Hungary; Centro de Investigación en Medicina Aplicada (CIMA), SPAIN

## Abstract

**Background & Aim:**

MicroRNAs (miRs) regulate hepatic steatosis, inflammation and fibrosis. Fibrosis is the consequence of chronic tissue damage and inflammation. We hypothesized that deficiency of miR-155, a master regulator of inflammation, attenuates steatohepatitis and fibrosis.

**Methods:**

Wild type (WT) and miR-155-deficient (KO) mice were fed methionine-choline-deficient (MCD) or -supplemented (MCS) control diet for 5 weeks. Liver injury, inflammation, steatosis and fibrosis were assessed.

**Results:**

MCD diet resulted in steatohepatitis and increased miR-155 expression in total liver, hepatocytes and Kupffer cells. Steatosis and expression of genes involved in fatty acid metabolism were attenuated in miR-155 KO mice after MCD feeding. In contrast, miR-155 deficiency failed to attenuate inflammatory cell infiltration, nuclear factor κ beta (NF-κB) activation and enhanced the expression of the pro-inflammatory cytokines tumor necrosis factor alpha (TNFα) and monocyte chemoattractant protein-1 (MCP1) in MCD diet-fed mice. We found a significant attenuation of apoptosis (cleaved caspase-3) and reduction in collagen and α smooth muscle actin (αSMA) levels in miR-155 KO mice compared to WTs on MCD diet. In addition, we found attenuation of platelet derived growth factor (PDGF), a pro-fibrotic cytokine; SMAD family member 3 (Smad3), a protein involved in transforming growth factor-β (TGFβ) signal transduction and vimentin, a mesenchymal marker and indirect indicator of epithelial-to-mesenchymal transition (EMT) in miR-155 KO mice. Nuclear binding of CCAAT enhancer binding protein β (C/EBPβ) a miR-155 target involved in EMT was significantly increased in miR-155 KO compared to WT mice.

**Conclusions:**

Our novel data demonstrate that miR-155 deficiency can reduce steatosis and fibrosis without decreasing inflammation in steatohepatitis.

## Introduction

MicroRNAs (miRNAs) are small non-coding RNAs that regulate the expression of numerous target genes at the transcriptional or translational level and play important roles in liver diseases [1]. Altered miRNA profiles were reported in patients with non-alcoholic steatohepatitis (NASH) compared to healthy controls [2,3], as well as in various animal models of NASH [4,5]. NASH is characterized by steatosis, inflammation, hepatocyte death and at later stages fibrosis, cirrhosis, and the development of hepatocellular carcinoma (HCC) [5]. All of these processes can be regulated by miRNAs [1].

A clinically relevant challenge in NASH research is to define factors that lead to progression of steatosis to steatohepatitis and fibrosis. Increasing evidence suggests the role of innate immunity, pattern recognition receptors, including TLR4 and TLR9, stimulated by various microbial and endogenous danger molecules in the development of steatohepatitis and fibrosis [6,7]. miRNA-155 (miR-155) is a master regulator of inflammation that affects both innate and adaptive immunity [8]. miR-155 is induced by Toll-like receptor (TLR) ligands and it enhances the translation of tumor necrosis factor alpha (TNFα), a pro-inflammatory cytokine identified in the pathogenesis of the metabolic syndrome and steatohepatitis [9]. Increased miR-155 has been found in the liver in a mouse model of alcoholic liver disease (ALD) in hepatocytes [10] and in Kupffer cells [11]. Furthermore, miR-155 is enhanced TNFα production in Kupffer cells in ALD [11]. Alcohol increased miR-155 in macrophages via NF-κB activation, and up-regulation of miR-155 was induced by the TLR4 ligand, lipopolysaccharide (LPS) in ALD [11,12]. Increased gut permeability, elevated serum endotoxin, and increased TNFα production by liver macrophages are causally related in the pathogenesis of both alcoholic [11] and non-alcoholic steatohepatitis [13]. There are several models of nonalcoholic steatohepatitis, with substantial differences [14]. Increased miR-155 expression has been reported in both the choline-deficient-amino acid defined (CDAA) and the high fat diet (HFD) NASH models [5,15]. However, its role in the methionine-choline deficient (MCD) model, particularly in inflammation and innate immune responses awaits investigation. To study inflammation and fibrosis, the MCD model has some advantages compared to other models, despite of the lack of peripheral insulin resistance. While HFD induces steatosis, the inflammation is less prominent, and there is no or minimal fibrosis compared to the MCD diet. The degree of necroinflammatory changes and fibrosis is more severe and rapid in MCD-steatohepatitis making it more suitable for studying the progression of NASH.

Persistent and excessive liver damage leads to chronic inflammation and fibrosis [16]. Impairment in the pathways involved in inflammation, tissue repair, and excessive deposition of extracellular matrix leads to liver fibrosis. Recruited inflammatory cells and resident macrophages, and Kupffer cells produce cytokines, including IL-1β, TGFβ, etc., that contribute directly or indirectly to the activation of hepatic stellate cells (HSCs) and therefore liver fibrosis [17].

Here we hypothesized that miR-155 has a role in the development and progression of nonalcoholic steatohepatitis and fibrosis. Our novel data show that miR-155 deficiency promotes inflammation, and increases some inflammatory cytokines/chemokines such as TNFα and MCP1 in MCD-steatohepatitis. This shows the complex role of miR-155 in the inflammatory pathways and also emphasizes the importance of its negative regulatory role in inflammation. Our findings also revealed that despite of the significant inflammation, miR-155 deficiency attenuates steatosis and fibrosis in NASH suggesting that miR-155 regulates fibrosis, at least partially, independent of inflammation in the liver.

## Materials and Methods

### Animal studies

This study was approved by the University of Massachusetts Medical School Institutional Animal Use and Care Committee. Six-to-eight week-old female C57Bl/6 wild type (WT) mice were fed with methionine-choline-deficient (MCD) diet for 3, 6 or 8 weeks; controls received a DL-methionine (3 g/kg) and choline bitartrate (2 g/kg) supplemented (MCS) diet *(Dyets Inc*., *Bethlehem*, *PA*, *USA)*; n = 5–10. miR-155 deficient (knock out/KO) mice with the appropriate control groups were fed with MCD or MCS diet for 5 weeks (n = 6–10). miR-155 KO mice were purchased from Jackson laboratory *(Bar Harbor*, *Maine*, *USA)* and breeding colony was maintained in the animal facility of UMMS.

### Liver cell isolation

Primary murine hepatocytes, liver mononuclear cells (LMNCs) and Kupffer cells (KCs) were isolated from MCS or MCD diet-fed mice by an enzyme-based tissue digestion method as described previously [18,11]. LMNCs and KCs were plated and stimulated or not with 100ng/ml LPS for 6 hours; TNFα protein levels were measured in the supernatants using ELISA *(BD Biosciences*, *San Jose*, *CA*, *USA)*, while cellular miR-155 expression was evaluated by qPCR as described [11].

### Biochemical analysis and cytokine measurements

Serum alanine aminotransferase (ALT) was determined using a kinetic method *(D-TEK*, *Bensalem*, *PA*, *USA)*. Liver triglyceride levels were assessed using the L-Type Triglyceride H kit *(Wako Chemicals USA Inc*., *VA*, *USA)*. Serum and liver TNFα *(BioLegend Inc*., *San Diego CA*, *USA)*, IL-1β *(R&D Systems*, *Minneapolis*, *MN*, *USA)* and MCP1 *(BioLegend Inc*., *San Diego CA*, *USA)* levels were determined by ELISA as described by manufactures.

### Histopathological analysis

Sections of formalin-fixed livers were stained with hematoxylin-eosin and scored for steatosis, necrosis and lobular and portal inflammation by a pathology expert using the scoring system described by Kleiner DE, Brunt EM et al. [19]. Fibrosis was assessed by Sirius Red staining and quantification of the Sirius Red positive area using Image J program. Oxidative stress was evaluated by 4-Hydroxynonenal (4-HNE) immunohistochemistry (IHC). The slides were analyzed under light microscopy at 100X and 200X.

### RNA analysis

Total RNA was extracted using the RNeasy kit *(Qiagen Sciences*, *Maryland*, *USA)*. cDNA was transcribed with Reverse Transcription System *(Promega Corp*., *Madison*, *WI)*. Real-time quantitative polymerase chain reaction was performed using iCycler *(Bio-Rad Laboratories Inc*., *Hercules*, *CA)* and SYBR Green. Primer sequences are available upon request. All results were normalized to 18S mRNA expression.

For miRNA analyses total RNA was isolated using Direct-zol RNA MiniPrep with on column DNA digestion *(Zymo Research Corp*., *Irvine*, *CA*, *USA)* and RT-qPCR were performed using TaqMan miRNA assays (Ambion, Austin, TX, USA); all results were normalized to snoRNA202 expression.

### Western blot

Whole cell lysates were extracted from liver. Samples with equal amounts of protein were separated in a polyacrylamide gel, transferred and identified on nitrocellulose membrane with specific primary antibodies followed by HRP–labeled secondary antibodies. The following antibodies were applied: αSMA *(Abcam*, *Cambridge*, *MA*, *USA)*; PDGF *(Abcam*, *Cambridge*, *MA*, *USA);*; caspase-3 *(Cell Signaling*, *Danvers*, *MA)*, Smad2/3 *(Cell Signaling*, *Danvers*, *MA)*. Beta actin or beta tubulin *(Abcam*, *Cambridge*, *MA*, *USA)* were used as loading controls.

### Electrophoretic Mobility Shift Assay (EMSA)

Liver nuclear proteins were isolated as described [20] and 5μg nuclear protein was subjected to EMSA using consensus, double-stranded HRE oligonucleotide specific for NFκB and C/EBP *(Santa Cruz Biotechnology*, *Santa Cruz*, *CA*, *USA)*. C/EBPβ nuclear binding was detected by EMSA supershift using C/EBPβ specific antibody *(Santa Cruz Biotechnology*, *Santa Cruz*, *CA*, *USA)* 30 min prior to the labeled oligonucleotide.

### Statistical analysis

Statistical significance was determined using the non-parametric Kruskal-Wallis and Mann-Whitney tests. Data are shown as mean±standard error and were considered statistically significant at p<0.05.

## Results

### MCD diet-induced steatohepatitis is associated with increased miR-155 expression in parenchymal and non-parenchymal cells in the liver

**Fig 1:**
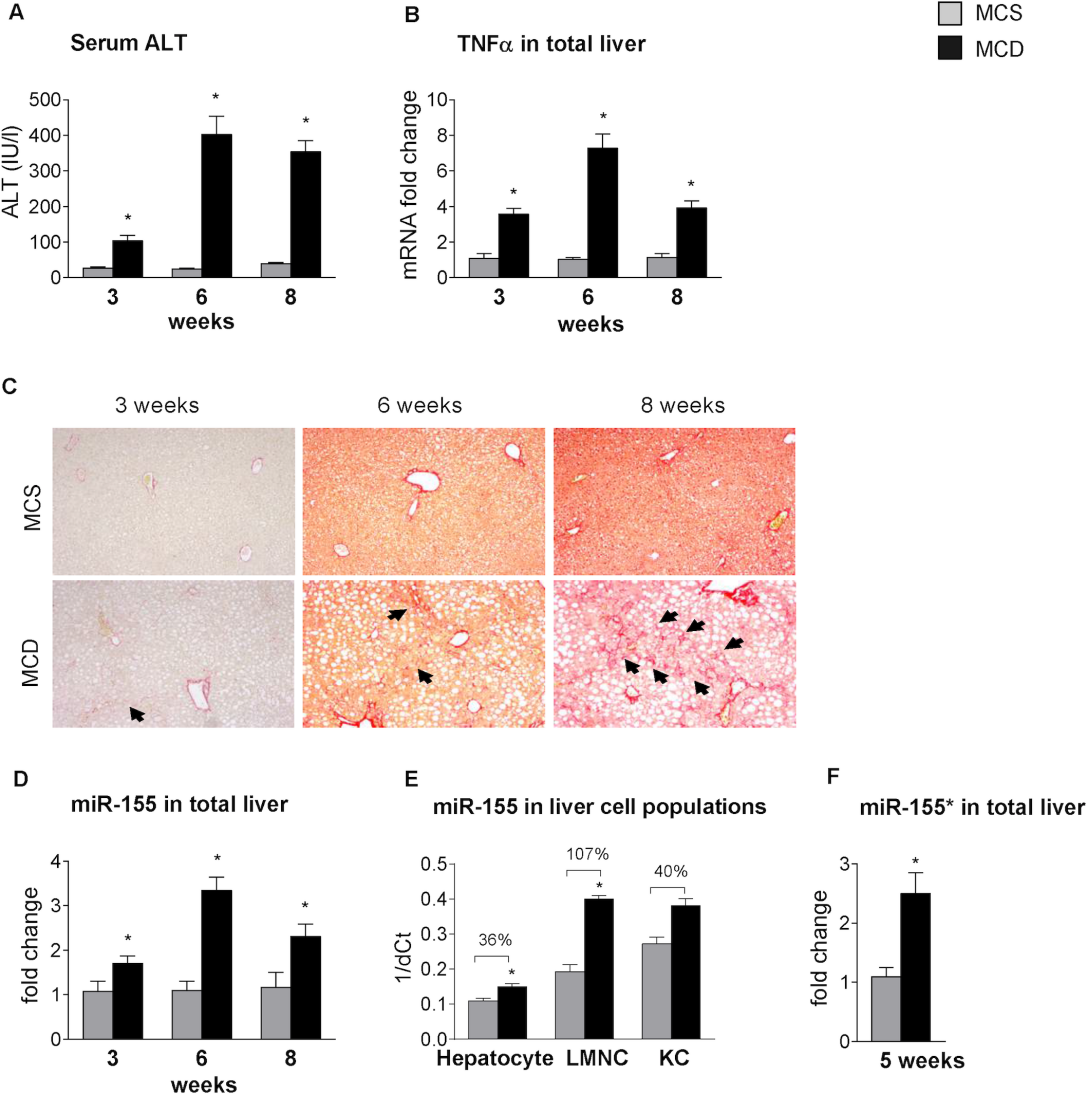
MCD diet-induced steatohepatitis is associated with increased miR-155 expression in parenchymal and non-parenchymal cells. C57Bl/6 mice were fed with methionine-choline deficient (MCD) or supplemented (MCS) control diet for 3, 6 and 8 weeks. Serum alanine aminotransferase levels (A) and TNFα mRNA expression (B) were determined (n = 5-8/group). Liver fibrosis was assessed by Sirius Red staining (100x; n = 3-6/group), representative slides are shown (C). miR-155 expression was detected by qPCR in total livers (n = 5-8/group) (D). Primary murine hepatocytes (n = 7/group), Kupffer cells (n = 6, pooled data, 2 datapoints/group) and liver mononuclear cells (LMNC; n = 3-4/group) were isolated from a subset of mice after 6 weeks of MCS or MCD diet feeding and cell-specific miR-155 expression was determined and represented as 1/dCt (E). miR-155* expression was determined in total livers (F). (*) indicates p<0.05 MCS vs. corresponding MCD group. Statistics was performed on fold change data.

In previous studies, the miRNA profile of steatohepatitis in various animal models [4,5] and in human non-alcoholic steatohepatitis (NASH) [2,3] has been investigated, but little is known about their functional role in the pathogenesis of the disease. The methionine-choline deficient model of steatohepatitis results in early lipid accumulation, prominent necro-inflammation and later fibrosis [21], as shown in Fig 1 (A: serum ALT, B: liver TNFα mRNA, C: Sirius Red staining). Therefore, despite its disadvantages, such as lack of peripheral insulin resistance, it is a useful tool to study the progression of steatohepatitis. MicroRNA-155 (miR-155), a master regulator of inflammation, enhances the translation of TNFα, a pro-inflammatory cytokine induced during innate immune responses by Toll-like receptor (TLR) ligands [8,9]. Increased miR-155 expression has been reported in choline-deficient-amino-acid-defined (CDAA) and in high fat diet models of steatohepatitis [5,15], but little is known in MCD diet-induced steatohepatitis. Here, we show increased miR-155 expression in the livers of MCD diet-fed mice throughout the progression of the disease, with a peak at 6 weeks (Fig 1D). miR-155 is abundantly expressed in immune cells [8], however, low expression is also present in hepatocytes [5,10]. Thus, we evaluated the cell-specific expression of miR-155 in isolated hepatocytes, liver resident macrophages (Kupffer cells [KCs] and liver mononuclear cells (LMNCs), the latter containing monocytes, lymphocytes and dendritic cells. The purity of these cell populations was previously reported [18]. We found increased miR-155 expression in LMNCs (107% increase over MCS) and in hepatocytes (36% increase over MCS) in MCD diet-induced steatohepatitis (Fig 1E). There was a 40% increase in miR-155 expression in KCs as well, but statistical significance could not be calculated due to pooled samples resulting in a small sample size (Fig 1E). The complementary strand miR-155* expression was also increased in the livers of MCD diet-fed mice (Fig 1F).

### miR-155 deficiency attenuates liver steatosis but does not prevent liver injury in MCD-induced steatohepatitis

**Fig 2:**
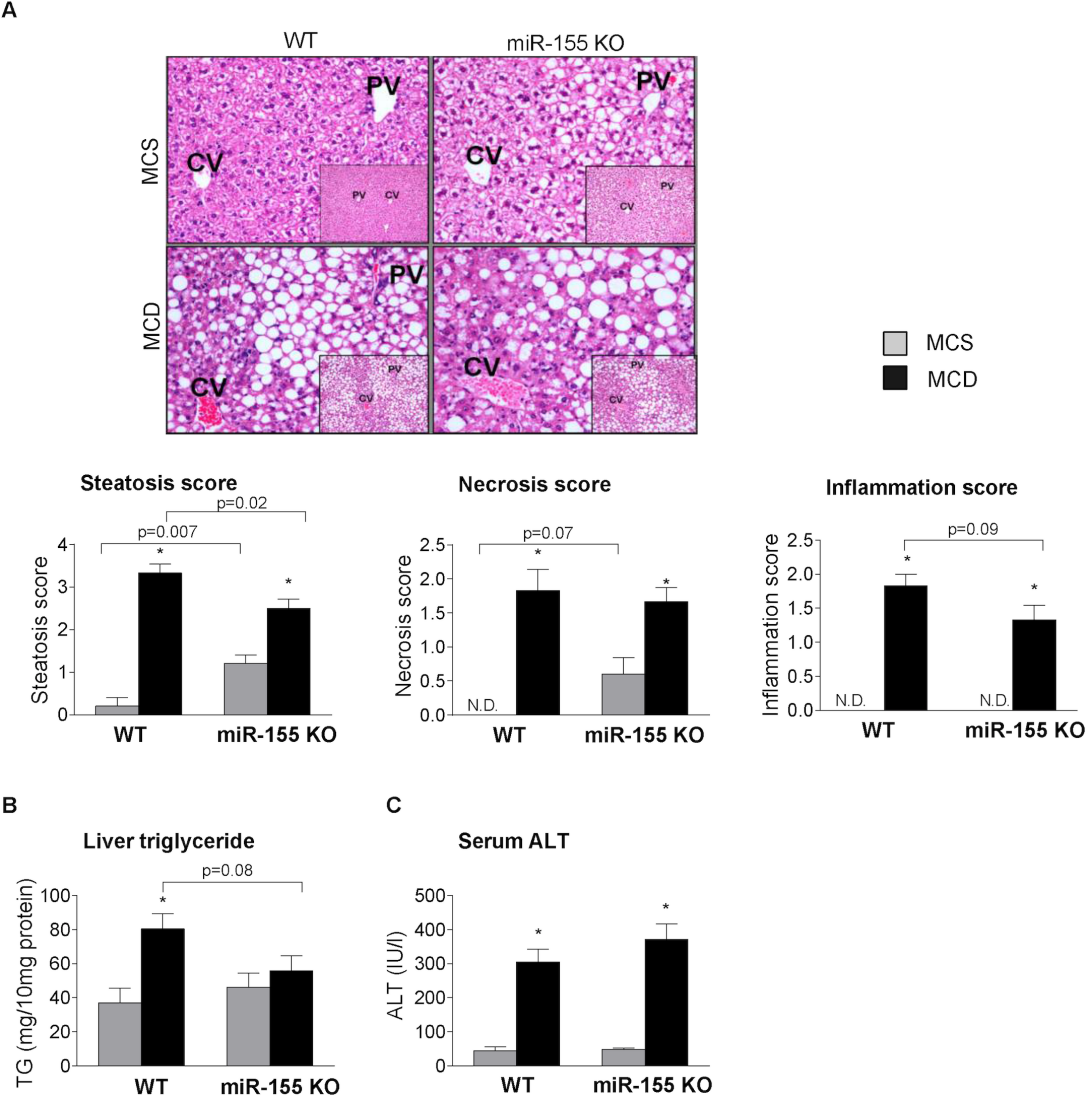
miR-155 deficiency does not prevent liver injury, but attenuates liver steatosis in MCD-steatohepatitis. Wild type (WT) and miR-155 deficient (KO) mice were fed with methionine-choline deficient (MCD) or supplemented (MCS) control diet for 5 weeks. Liver histology was evaluated by hematoxilin-eosin staining (200x, inserts 100x; n = 5/group), representative slides are shown (A). Steatosis, necrosis, and lobular inflammation, were scored by a pathology expert (A). Liver triglyceride (B) and serum ALT (C) levels were determined (n = 6-8/group). (*) indicates p<0.05 MCS vs. corresponding MCD group. N.D. = not detectable or score = 0.

To study the biological role of miR-155 in steatohepatitis in vivo we used miR-155 deficient mice. We found attenuated hepatic steatosis in the miR-155 deficient mice compared to the WTs after MCD diet feeding at 5 weeks (Fig 2A: liver histology steatosis score, 2B: liver triglyceride levels). In contrast, in the MCS diet-fed control mice miR-155 deficiency enhanced the fat deposition compared to WTs (Fig 2A: liver histology steatosis score, 2B: liver triglyceride levels). Liver inflammation and injury indicated by histology inflammation score, necrosis score (Fig 2A), and serum ALT (Fig 2C) were not significantly attenuated by miR-155 deficiency in MCD diet-induced steatohepatitis. Notably, serum ALT levels were increased at an interim time point (2 weeks; data not shown) similar to that found in and earlier high fat diet feeding [15].

The different effect of miR-155 deficiency on steatosis between MCD and MCS diet-fed control mice suggests that miR-155 might have different role/s in fat accumulation in normal and diseased conditions, and its effect might depend on the major pathogenetic steps of steatosis, such as increased fatty acid influx and / or synthesis vs. “lipid trap”. The latter is involved in the development of MCD-steatohepatitis due to the decreased phosphatidylcholine and very low density lipoprotein (VLDL) synthesis [14].

Therefore next we analyzed the expression of genes involved in (1) fatty acid uptake (adipose differentiation-related protein / Adrp); (2) triglyceride synthesis (diacylglycerol O-acyltransferase 2 / Dgat2); (3) fatty acid synthesis (acetyl-Coenzyme A carboxylase / Acc1; fatty acid synthase / Fasn); (4) fatty acid oxidation (carnitine palmitoyl transferase 1a / Cpt1a); (5) fatty acid binding protein (Fabp4) that links lipid metabolism with inflammation; (6) cholesterol metabolism (low density lipoprotein receptor / Ldlr; 3-hydroxy-3-methylglutaryl-CoA-reductase / Hmgcr); and (7) transcription factors involved in lipid metabolism (nuclear receptor subfamily 1, group H, member 3 / Nr1h3 / LXRα; peroxisome proliferator activated receptor alpha / Ppara).

**Fig 3:**
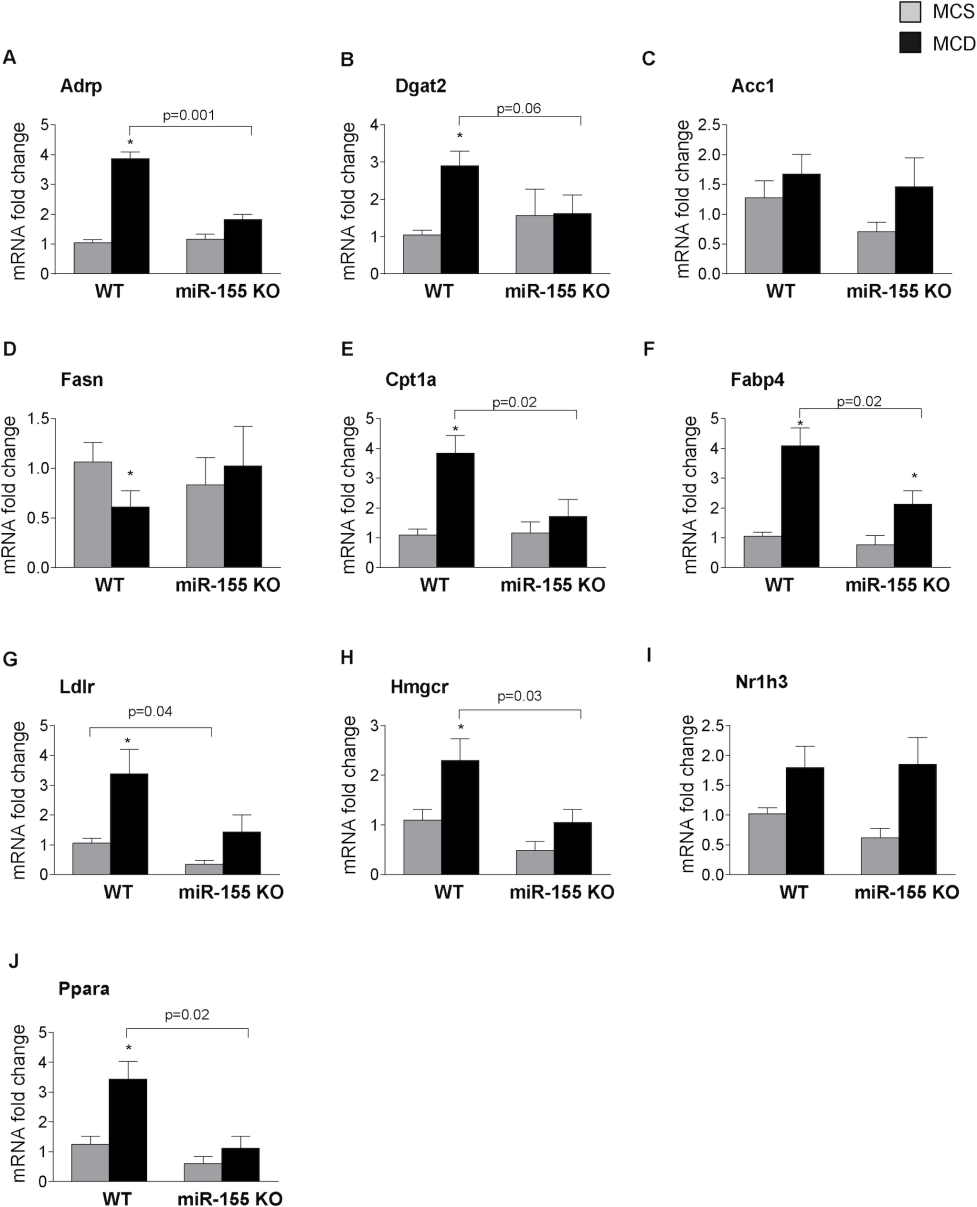
miR-155 deficiency alters expression of genes in the lipid metabolism. Wild type (WT) and miR-155 deficient (KO) mice were fed with methionine-choline deficient (MCD) or supplemented (MCS) control diet for 5 weeks. mRNA expression of Adrp (A), Dgat2 (B), Acc1 (C), Fasn (D), Cpt1a (E), Fabp4 (F), Ldlr (G), Hmgcr (H), Nr1h3 (I) and Ppara (J) was measured in the livers (n = 6-8/group). (*) indicates p<0.05 MCS vs. corresponding MCD group.

Adrp, a lipid droplet protein that promotes fatty acid storage in form of triglycerides and inhibits VLDL secretion [22] and Dgat2, another protein facilitating triglyceride synthesis [23] were increased in WT MCD fed mice and this increase was prevented in miR-155 deficient mice (Fig 3A and 3B). While we found no significant difference between genotypes (WT and miR-155 KO) in the expression of Acc1 (Fig 3C) (a gene involved in fatty acid synthesis) the MCD diet induced significant reduction of Fasn expression that was rescued by mir-155 deficiency (Fig 3D). In concordance with previous studies, Cpt-1a, a key rate-limiting enzyme in the fatty acid oxidation [24] was increased in MCD-steatohepatitis in WT mice and not in miR-155 KO mice (Fig 3E). Fabp4, an adipokine that links lipid metabolism and inflammation [25], was increased by MCD diet in WT mice, and was attenuated by miR-155 deficiency (Fig 3F). Ldlr, a cell surface receptor responsible for the cellular uptake of low density lipoprotein (LDL) molecule [26], was significantly increased in MCD diet-fed mice compared to MCS controls. miR-155 deficiency attenuated Ldlr expression in both MCS and MCD groups, however in the latter, it did not reach statistical significance (p = 0.07) (Fig 3G). Similarly, Hmgcr, the rate limiting enzyme in cholesterol synthesis was increased in the MCD diet-fed WT mice, and miR-155 deficiency attenuated Hmgcr expression, which reached statistical significance in the MCD diet-fed group (Fig 3H) [27]. In contrast to the report on miR-155 deficiency in HFD [15], we did not find an increased expression of the transcription factor Nr1h3 (LXRα) in miR-155 KO mice (Fig 3I). This might explain the different findings on some LXR-reponsive genes (Fasn–Fig 3D, CD36- data not shown). PPARα, another transcription factor involved in lipid metabolism [28], was highly induced by MCD diet in WT mice, and the increase was completely prevented in the miR-155 KOs (Fig 3J).

In summary, miR-155 deficiency in MCD diet-fed mice affected Adrp, Dgat2, Fasn, Cpt1a, Fabp4, Hmgcr and Ppara expression, while in MCS diet-fed controls Ldlr expression was significantly attenuated. The decreased Ldlr levels might result in impaired Ldl clearance and as a consequence of this, higher cholesterol levels [26]. Since cholesterol levels correlate with the intrahepatic fat content [29], it is tempting to speculate that the higher cholesterol levels might potentially contribute to the development of steatosis in the miR-155 deficient MCS diet-fed mice.

### miR-155 deficiency attenuates liver fibrosis in MCD-induced steatohepatitis

**Fig 4:**
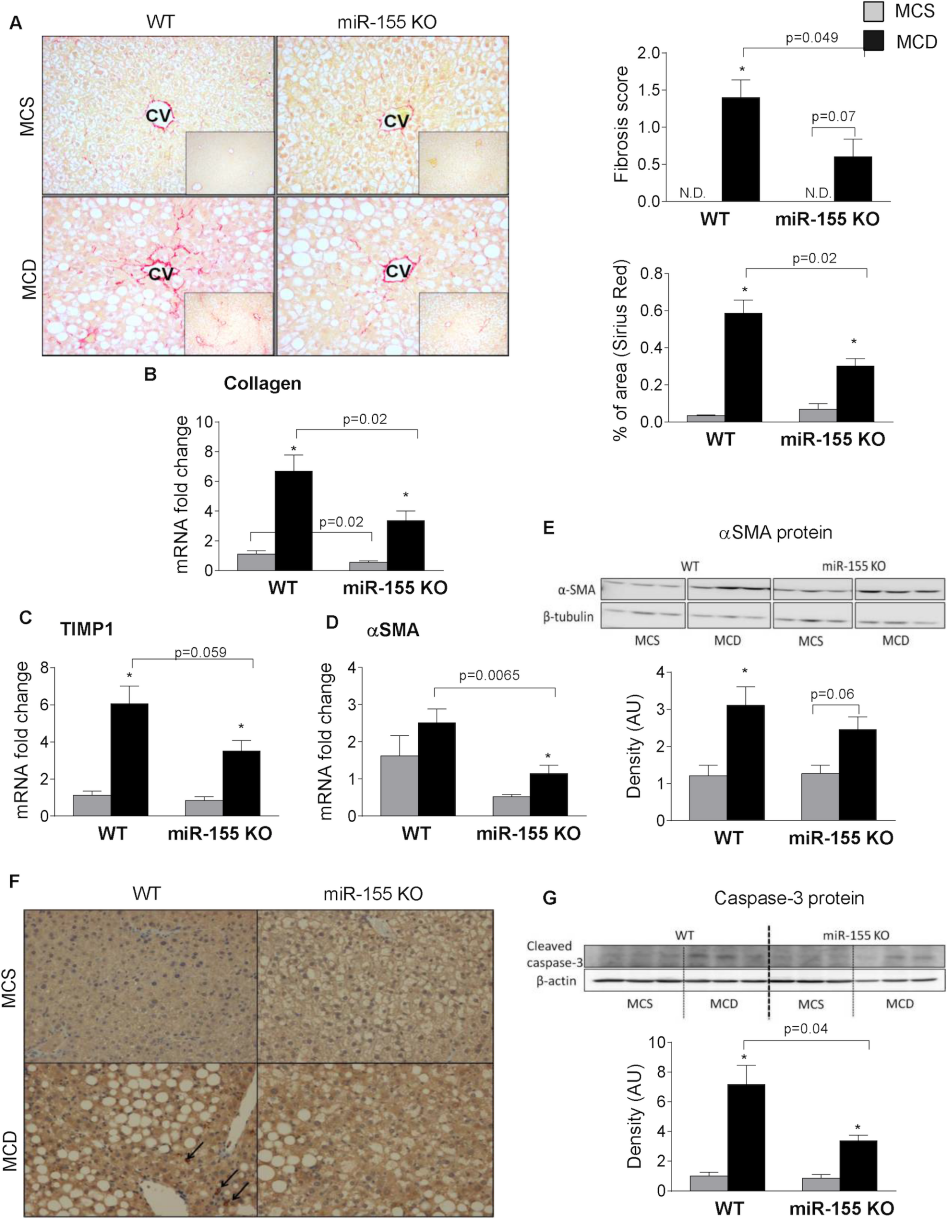
miR-155 deficiency attenuates liver fibrosis in MCD-steatohepatitis. Wild type (WT) and miR-155 deficient (KO) mice were fed with methionine-choline deficient (MCD) or supplemented (MCS) control diet for 5 weeks. Liver fibrosis was assessed by Sirius Red staining (200x; n = 3-5/group), representative slides are shown. Fibrosis was scored by an expert pathologist as well as quantified using Image J program (A, right panels). Liver collagen-1a (B), TIMP-1 (C) and αSMA (D) mRNA expression was measured by qPCR (n = 6-8/group). αSMA protein expression was detected by Western blot from whole liver lysates (E, top panel: Western blot; E, bottom panel: densitometry; n = 3/group). Oxidative stress was evaluated by 4-HNE IHC (200x; n = 3-5/group), representative slides are shown (F). Caspase-3 protein expression was assessed by Western blot (G, top panel: Western blot; G, bottom panel: densitometry; n = 3/group). (*) indicates p<0.05 MCS vs. corresponding MCD group. N.D. = not detectable or score = 0.

MCD diet results in liver fibrosis; therefore, next we evaluated the effect of miR-155 deficiency on fibrosis. Despite comparable liver injury and inflammation, (Fig 2A) we found significantly reduced liver fibrosis in the miR-155 deficient mice after MCD diet feeding indicated by the Sirius Red staining and fibrosis score (Fig 4A). Genes related to fibrosis, such as collagen 1a (Fig 4B), tissue inhibitor of metalloproteinase 1 (TIMP-1) (Fig 4C) and αSMA (Fig 4D) were reduced in the miR-155 deficient mice compared to the WTs after MCD feeding. The SMA protein expression was significantly increased only in WT mice and not in miR-155 KO mice after MCD diet (Fig 4E). Previously, others and we have shown the role of oxidative stress in the development of fibrosis in non-alcoholic steatohepatitis [6,30]. Here, we found that MCD diet resulted in increased 4-HNE staining, a marker of oxidative stress, in both genotypes compared to the MCS control groups (Fig 4F). Furthermore, it appears that MCD diet-fed WT animals had more 4-HNE adducts (black arrows) than the MCD diet-fed miR-155KOs suggesting a potential role of oxidative stress in fibrosis. Oxidative stress also plays a pivotal role in cell death (apoptosis); a link to stellate cell activation [31]. Here, we found increased cleaved (active) caspase-3 expression in the MCD diet-fed WT mice compared to MCS controls and it was significantly attenuated in the miR-155 KOs on MCD diet (Fig 4G). This suggests less apoptotic cell death in the miR-155 KO animals, despite the overall comparable liver injury.

### miR-155 deficiency does not prevent inflammation in MCD-induced steatohepatitis

**Fig 5:**
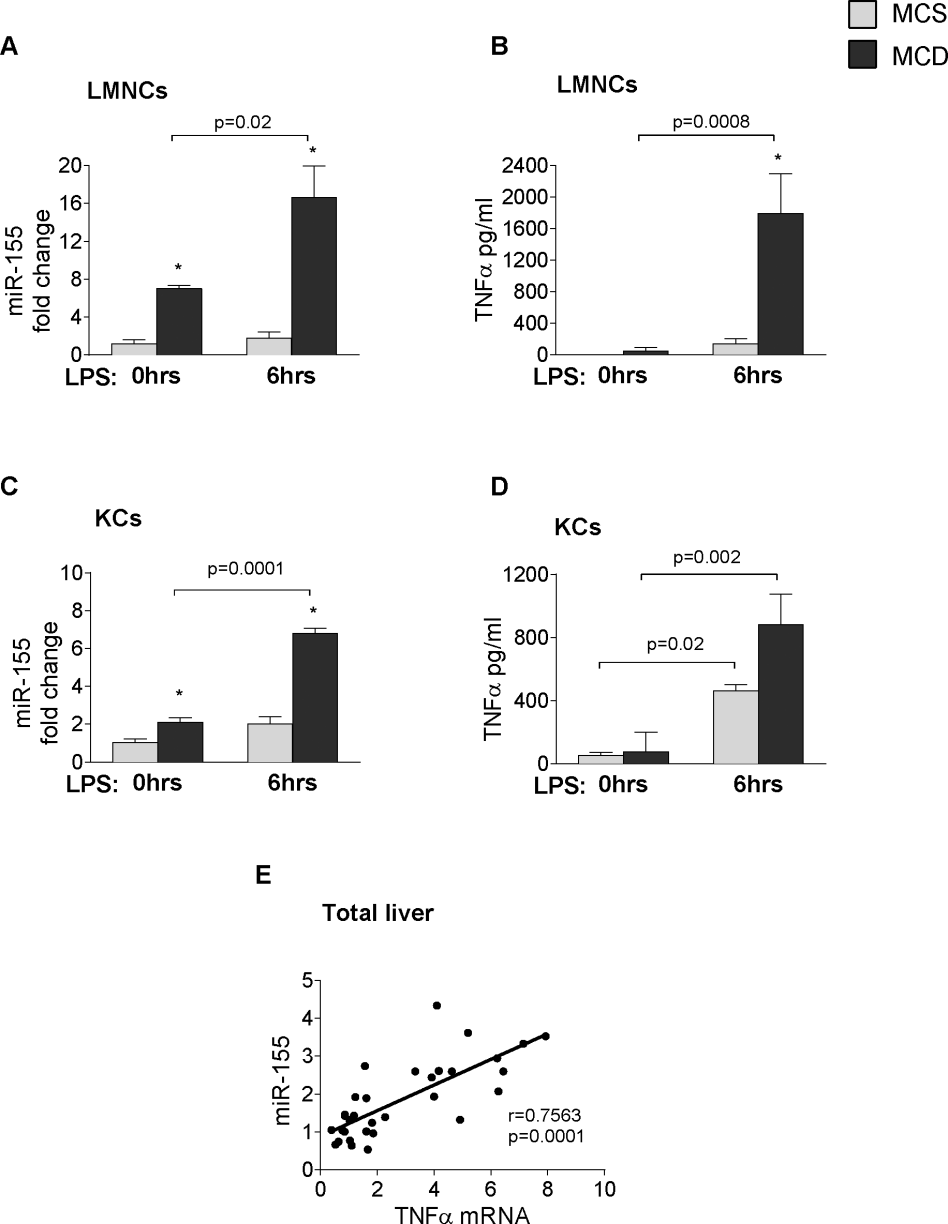
LPS induces miR-155 and TNFα expression in hepatic immune cells. Isolated LMNCs and Kupffer cells from C57Bl/6 WT mice were stimulated or not with 100ng/ml LPS for 6 hours in vitro. miR-155 expression (A: LMNCs, C: KCs) and TNF-α protein secretion (B: LMNCs, D: KCs) were measured in the cells and in the supernatant, respectively (n = 8-10/group). RNA was isolated from C57Bl/6 WT mice fed with methionine-choline deficient (MCD) or supplemented (MCS) control diet for 3, 6 and 8 weeks. miR155 and TNFα mRNA expression was determined (n = 5-6/group, total 32) and correlation was plotted (E); correlation coefficient is shown. (*) indicates p<0.05 MCS vs. corresponding MCD group.

Liver fibrosis is preceded by inflammation in NASH [16]. miR-155, a master regulator of inflammation, is induced by TLR ligands and enhances the translation of TNFα [11]; a pro-inflammatory cytokine identified in the pathogenesis of metabolic syndrome and steatohepatitis [6]. Thus next we studied hepatic inflammation and evaluated the role of TLR activation in miR-155 and TNFα expression. An in vitro challenge with the TLR4 ligand LPS, induced a significantly higher miR-155 expression and TNFα secretion in isolated liver mononuclear cells (LMNCs) or Kupffer cells (KCs) in MCD-steatohepatitis compared to MCS controls (Fig 5A: LMNCs–miR-155, Fig 5B: LMNCs–TNFα; Fig 5C: KCs–miR-155, Fig 5D: KCs–TNFα). Hepatic miR-155 expression also showed a positive correlation with TNFα mRNA in the WT livers (Fig 5E).

**Fig 6:**
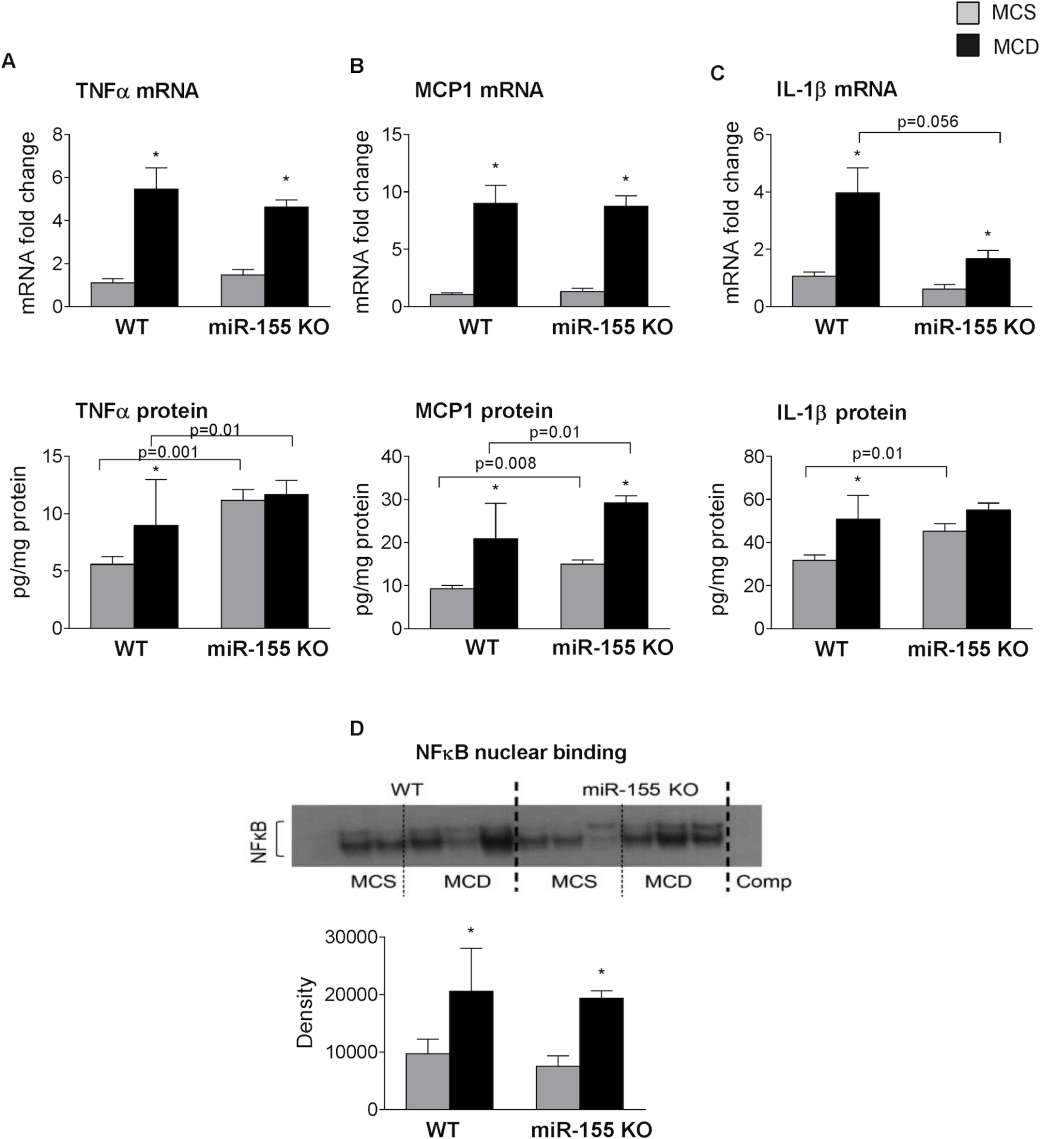
miR-155 deficiency does not attenuate hepatic inflammation in MCD-steatohepatitis. Wild type (WT) and miR-155 deficient (KO) mice were fed with methionine-choline deficient (MCD) or supplemented (MCS) control diet for 5 weeks. Liver TNFα (A top panel: mRNA, A bottom panel: protein), MCP1 (B top panel: mRNA, B bottom panel: protein) and IL1-β (C top panel: mRNA, C bottom panel: protein) mRNA and protein levels were measured by qPCR and ELISA, respectively (n = 6-8/group). NF-κB nuclear binding was evaluated by EMSA using liver nuclear extracts (D, top panel: representative blot, bottom panel: densitometry showing cumulative data of n = 6/group). (*) indicates p<0.05 MCS vs. corresponding MCD group.

However, despite the positive correlation between miR-155 and TNFα levels, we found that overall, hepatic inflammation was not attenuated in the miR-155 deficient mice (Fig 2). miR-155 deficiency did not reduce TNFα mRNA or protein levels (Fig 6A, mRNA upper panel, protein lower panel), and TNFα protein levels were higher in the miR-155 KO mice (Fig 6A lower panel). Monocyte chemoattractant protein (MCP1), one of the key chemokines was also comparable between the genotypes at the mRNA level, and higher protein levels were found in the miR-155 deficient mice (Fig 6B, mRNA upper panel, protein lower panel). IL-1β, a pro-inflammatory and pro-fibrotic cytokine that is a putative miR-155* target, was reduced at mRNA level in miR-155 KOs compared to WTs on MCD diet (Fig 6C upper panel). MCD diet induced increased IL-1β protein (total) only in WT mice, not in the miR-155 KOs (Fig 6C lower panel) compared to MCS controls. This might be related to the baseline IL-1β level in the miR-155 KO MCS control group.

Similarly to the cytokines, NF-κB nuclear binding was increased in both WT and miR-155 KO mice after MCD feeding (Fig 6D) suggesting a comparable level of inflammation in both genotypes.

### miR-155 regulates Smad3 and C/EBPβ activation in steatohepatitis induced liver fibrosis

**Fig 7:**
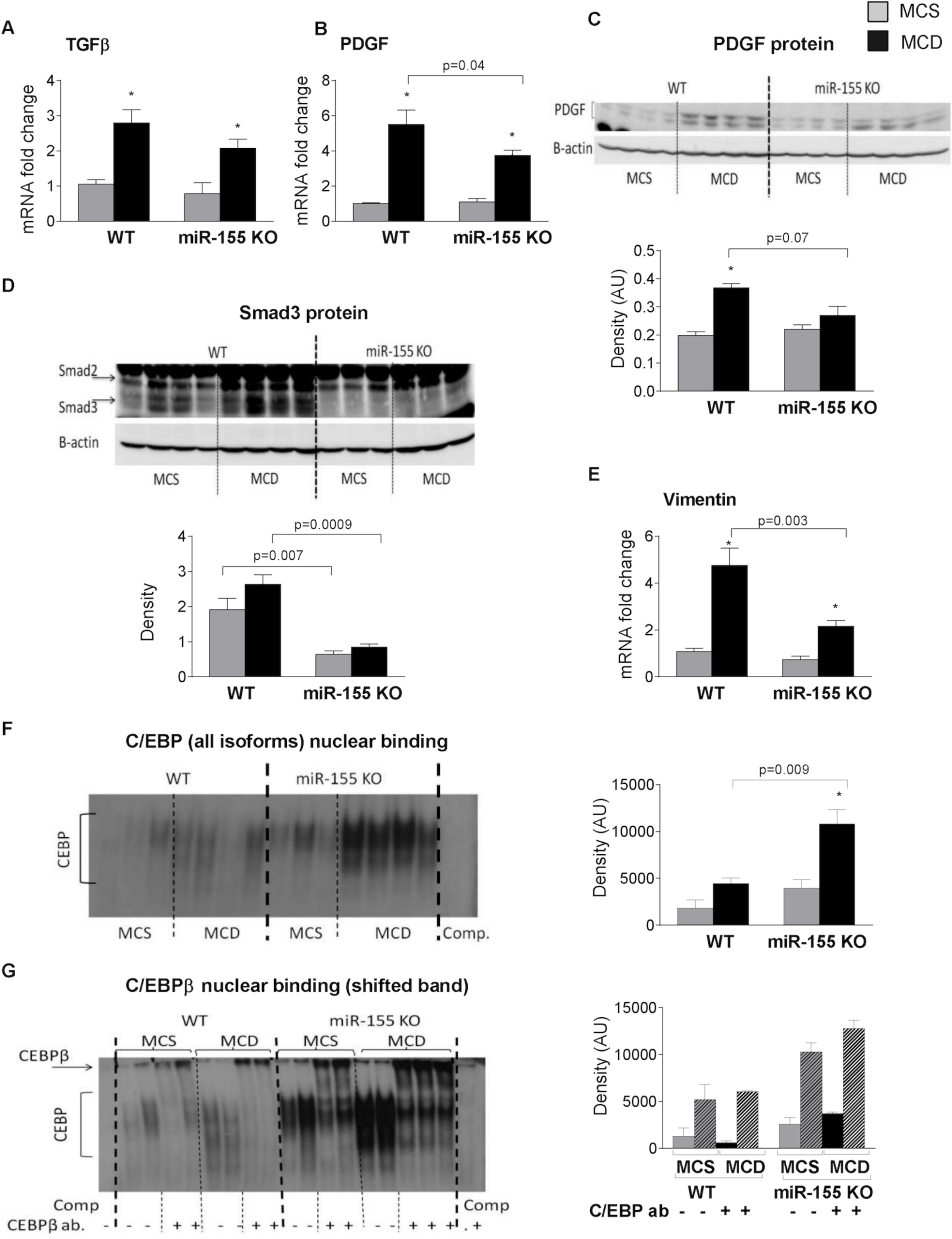
miR-155 deficiency attenuates Smad3 and vimentin expression and enhances C/EBPβ nuclear binding in MCD-steatohepatitis. Wild type (WT) and miR-155 deficient (KO) mice were fed with methionine-choline deficient (MCD) or supplemented (MCS) control diet for 5 weeks. Liver TGFβ (A) and PDGF (B) mRNA expression was measured by qPCR (n = 6–8). Liver PDGF (C) Smad2 and Smad3 protein expression (D) were analyzed by Western blot (top panels: blot, bottom panels: densitometry; n = 3-4/group). Vimentin (E) mRNA expression was measured by qPCR (n = 6-8/group). C/EBP nuclear binding was measured by EMSA using liver nuclear extracts (F, left panel: representative blot; right panel: densitometry, n = 6-8/group). C/EBPβ nuclear binding was assessed by EMSA supershift using C/EBPβ antibody (G, left panel), densitometry of the shifted band is graphed (G, right panel; n = 2/group). (*) indicates p<0.05 MCS vs. corresponding MCD group.

While inflammation can promote fibrosis, some studies have shown the divergence of these processes [21]. As a next step, we aimed to investigate how miR-155 affects fibrosis without significant attenuation of inflammation. Transforming growth factor β (TGFβ) and Platelet derived growth factor (PDGF), released by macrophages and liver sinusoidal endothelial cells, regulate hepatic stellate cell activation [17]. Previous studies have suggested a role for TGFβ in the development of fibrosis in NASH [6,7]. In our experiments there was no significant difference in TGFβ mRNA expression between WT and miR-155 KO mice both in the control and MCD groups (Fig 7A). However, PDGF, another pro-fibrogenic factor, was significantly attenuated in miR-155 deficient mice compared to WT mice both at the mRNA and protein levels (Fig 7B: mRNA and Fig 7C: protein) suggesting a potential role in the fibrosis development. While TGFβ mRNA levels were comparable between genotypes, there are several genes in TGFβ signaling that are putative mir-155 targets. Thus, next we evaluated some of the downstream signaling molecules of TGFβ such as the miR-155 target Smad2 [32] and Smad3 (www.microrna.org). Interestingly, we found no difference in Smad2 protein levels between genotypes (Fig 7D). However, we found a drastic reduction of Smad3 protein levels in the miR-155 KO animals (Fig 7D) suggesting that dysfunctional TGFβ signaling might contribute to the attenuated fibrosis in miR-155 KO mice.

We also evaluated markers of epithelial-mesenchymal transition (EMT), including E-cadherin and the mesenchymal marker, vimentin, as another possible mechanism of fibrosis. While there was no significant difference in the E-cadherin levels between the genotypes (data not shown), vimentin mRNA expression was significantly attenuated in the miR-155 KO mice compared to WTs after MCD diet feeding (Fig 7E). C/EBPβ is a target of miR-155 and recent studies have suggested a role of C/EBPβ in EMT [33,34]. We found enhanced C/EBP nuclear binding in the livers of miR-155 KO compared to WT mice after MCD feeding (Fig 7F). C/EBP exits in various forms, α, β, γ, δ [35], therefore next we determined the nuclear binding of C/EBPβ using a super shift assay. We found consistently enhanced C/EBPβ nuclear binding in miR-155 KO mice compared to WT mice after MCD feeding (Fig 7G). This data suggests that the lack of miR-155 expression promotes C/EBPβ activation in steatohepatitis.

## Discussion

Due to the increasing prevalence of obesity and metabolic syndrome, non-alcoholic fatty liver disease (NAFLD) [which includes simple steatosis (NAFL) and steatohepatitis (NASH)] is one of the most common liver diseases worldwide [36]. NASH is characterized by steatosis, hepatocellular injury and necroinflammation that might lead to fibrosis, cirrhosis and potentially hepatocellular carcinoma [37]. microRNAs, small non-coding RNAs, regulate lipid metabolism, inflammation, cell proliferation and regeneration at the transcriptional or translational level [1]. An altered miRNA profile has been reported in steatohepatitis in humans [2,3], as well as in animal models [4,5,38].

Here we report novel findings on the dual role of miR-155 on regulating steatosis and fibrosis in the methionine-choline deficient (MCD) model of steatohepatitis. We show that there is an increased miR-155 expression in both parenchymal and non-parenchymal cells in MCD diet-fed livers and we demonstrate that miR-155 deficiency attenuates steatosis in the MCD model. Our findings reveal that miR-155 regulates fibrosis at least partially independent of inflammation in NASH, since miR-155 deficiency attenuated MCD diet-induced fibrosis despite of sustained significant inflammation and liver injury. Finally, we propose that miR-155 affects fibrosis at multiple levels, via direct and indirect targets, including PDGF, Smad3 and C/EBPβ in NASH.

In the HFD model, hepatic steatosis was significantly enhanced by miR-155 deficiency [15]. Interestingly, here we found attenuated hepatic steatosis in the miR-155 deficient mice after the MCD diet. In contrast, mice on the MCS diet showed enhanced fat deposition similar to the previous study on HFD [15]. The different effect of miR-155 deficiency on steatosis in the MCD and MCS diets and the discrepancy between the MCD and HF diets suggest that miR-155 might have different roles in fat accumulation in healthy livers and in the various stages of diseased conditions. We found decreased steatosis at 5 weeks in MCD diet-fed miR-155 KO mice where liver fibrosis was also present. In contrast, the HFD model results in early steatosis with minimal or no fibrosis. Clinical observations suggest that the stage of NASH influences steatosis since reduced fat accumulation has been found in the fibrotic stage of NASH [39].

Increased fatty acid uptake, increased fatty acid synthesis, impaired fatty acid oxidation or impaired VLDL secretion might all contribute to hepatic steatosis [14]. While in a HFD model, one of the major mechanisms is the increased fatty acid influx, in MCD-steatohepatitis the methionine and choline deficiency leads to decreased phosphatidylcholine, and therefore VLDL synthesis [14], resulting a “lipid trap”. Here we found that miR-155 deficiency attenuated PPARα expression, a transcription factor involved in lipid metabolism [28]. Genes related to fatty acid uptake, storage and VLDL synthesis, including Adrp and Dgat2, as well as Cpt1a and Fabp4, all under potential PPARα regulation [40,41,42,43], were significantly reduced in miR-155 KO mice on MCD diet compared to WTs. Adrp promotes fatty acid storage in the form of triglycerides and inhibits VLDL secretion [22], and Dgat2 facilitates triglyceride synthesis [23]. Therefore, our findings suggest that miR-155 targets critical steps in the fatty acid uptake/TG synthesis/VLDL secretion, most likely indirectly, rather than the fatty acid synthesis, since we did not find significant differences between genotypes in the latter one. Notably, genes in fatty acid synthesis, such as Fasn, are under LXRα regulation [15], which was not affected by miR-155 deficiency in our model.

Beyond fatty acid metabolism, dietary cholesterol has also been shown to contribute to the development of fatty liver and steatohepatitis [29,44,45]. NAFLD is associated with dyslipidemic profile, including high large VLDL, small dense LDL and low HDL concentration [29]. Key molecules in cholesterol metabolism, Ldlr (LDL uptake / clearance) and Hmgcr (rate-limiting enzyme in cholesterol synthesis) were attenuated by miR-155 deficiency in HFD model [15]. Here, we found increased Ldlr and Hmgcr mRNA expression in MCD diet-fed WT mice compared to MCS controls. miR-155 deficiency significantly attenuated the Ldlr levels in the MCS control group, and to a lesser extent in the MCD diet-fed mice. We did not the have opportunity to measure cholesterol levels, but the reduced Ldlr expression might result in impaired LDL clearance and therefore higher cholesterol levels [26]. Therefore, it is tempting to speculate that the higher cholesterol levels are due to the impaired clearance, and play role in the augmented steatosis in the MCS control group. Following the logic of this hypothesis, the changes in Hmgcr might be compensatory. PPARα can regulate Ldlr transcription [28], and here we found that the MCD diet induced a significant increase in Ppara expression, which was prevented by miR-155 deficiency. Other genes that were affected by mir-155 deficiency in the HFD model (CD36, Cebpa, Pck1), have not changed in our MCD model.

Overall these results suggest that miR-155 targets lipid metabolism via multiple mechanisms and it might vary depending on the model of steatohepatitis. Further studies are needed to clarify the exact pathomechanism via which miR-155 targets genes in the lipid metabolism.

Hepatic steatosis is a risk factor; a preceding step for nonalcoholic steatohepatitis and its progression to fibrosis according to the two-hit hypothesis model [46]. However, here we found attenuation of steatosis, but not liver injury or inflammation in the miR-155 KO animals. Previous studies suggest that triglyceride accumulation could be protective against progressive liver damage, since DGAT2 inhibition resulted in reduced triglyceride synthesis but enhanced hepatic free fatty acids and oxidative stress [47]. While here we found reduced DGAT2 expression in miR-155 KO mice, there was no increase in oxidative stress compared to the WTs making this a less likely explanation in our model. MCD diet-fed WT mice seemed to have slightly more 4-HNE adducts. Oxidative stress plays pivotal role in cell death, a link to stellate cell activation [31]. Parallel with the MCD diet-induced enhanced oxidative stress, we found evidence of increased apoptosis in both WT and miR-155 KO mice on the MCD diet. However, there was a significant reduction in cleaved, active, caspase-3 levels in the miR-155 KOs compared to WTs on MCD diet, suggesting an attenuation of apoptosis by miR-155 deficiency despite the comparable overall liver injury (ALT levels). Cell death, including apoptosis is a feature of chronic liver diseases, and is associated with fibrosis [31]. Therefore, our data supports the hypothesis that the mechanisms of hepatocyte death (eg. apoptosis vs. necrosis) rather than simply the extent of it determine the fibrogenic response as it was suggested by Witek et al. [48].

Steatohepatitis, and in general any type of chronic inflammation and cell death, tissue damage of the liver, potentially leads to liver fibrosis. One of the proposed mechanisms of the fibrosis development is the monocyte/macrophage recruitment/activation and inflammatory and fibrogenic cytokine production [49,50]. The miR-155 target TNFα has been shown to enhance hepatic stellate cell activation and promote fibrosis in some studies [51]. However, here we found comparable inflammatory cell infiltration between genotypes and the TNFα and MCP1 protein levels were higher in the miR-155 KO mice. In some ways, this data is not surprising since miR-155 targets several negative and positive regulators of the inflammatory pathways [16]. In other ways though, it suggests the detachment of inflammatory cell infiltration and liver injury from fibrosis. Our observation is in concordance with some previous reports showing divergence of hepatic inflammation, injury and fibrosis [21,48].

According to previous studies IL-1β and TNFα influences fibrosis via hepatic stellate cell survival and not activation [52], while other cytokines, including TGFβ and PDGF, released by macrophages and liver sinusoidal endothelial cells, regulate hepatic stellate cell activation [17]. We found no significant difference in the TGFβ mRNA levels between genotypes either in the control or the MCD group. TGFβ is synthesized as a long precursor polypeptide that is cleaved to mature protein and Latency Associated Polypeptide (LAP). The bioactivity of mature TGFβ is dependent on its release from LAP. The measurement of bioactive TGFβ level in tissues is challenging. However, the drastic reduction of Smad3, a miR-155 target and downstream signaling protein of TGFβ suggests impaired TGFβ signaling, rather than impaired TGFβ levels in the miR-155 KO mice. Similarly, PDGF expression was also attenuated in the miR-155 KO animals. Overall, these suggest that miR-155 might contribute to liver fibrosis via activation of hepatic stellate cells in our model. The role of NADPH oxidase complex-dependent oxidative stress has also been reported in the activation of hepatic stellate cells [53]. Based on our data it appears that MCD diet induced oxidative stress, indicated by the 4-HNE staining, might be slightly higher in the WT animals compared to miR-155 KOs.

Stellate cells and their transdifferentiation into extracellular matrix producing myofibroblasts is the central event in fibrosis, however, other contributors of fibrogenic cells exist too, including portal fibroblasts, bone marrow-derived mesenchymal cells and EMT [17]. During EMT, epithelial cells undergo morphological changes to acquire a fibroblast-like phenotype with down-regulation of adhesion molecules and up-regulation of mesenchymal markers such as vimentin [54]. Reports are somewhat contradictory in terms of hepatocyte EMT [54,55]. Our data on the increased expression of vimentin raise the possibility of EMT. More importantly, we found a significantly decreased vimentin expression in the miR-155 KO mice. While vimentin is not a direct target of miR-155, C/EBPβ, a well-established miR-155 target [5] plays a role in EMT in various cancers [33,34]. Since a loss of C/EBPβ promotes EMT in mammary epithelial cells [33], it is tempting to speculate that augmentation of C/EBPβ in miR-155 KO mice, as we have found, might also contribute to an attenuation of fibrosis in our model. The regulation of fibrosis by miR-155 likely occurs at multiple levels, and it is not restricted to NASH-fibrosis; for example, we also found a reduction of carbon-tetrachloride induced-fibrosis in miR-155 deficient mice [56]. In addition to miR-155, numerous other microRNAs can affect liver fibrosis, including miR-122. We recently reported a direct link between miR-122 and vimentin expression [57].

**Fig 8:**
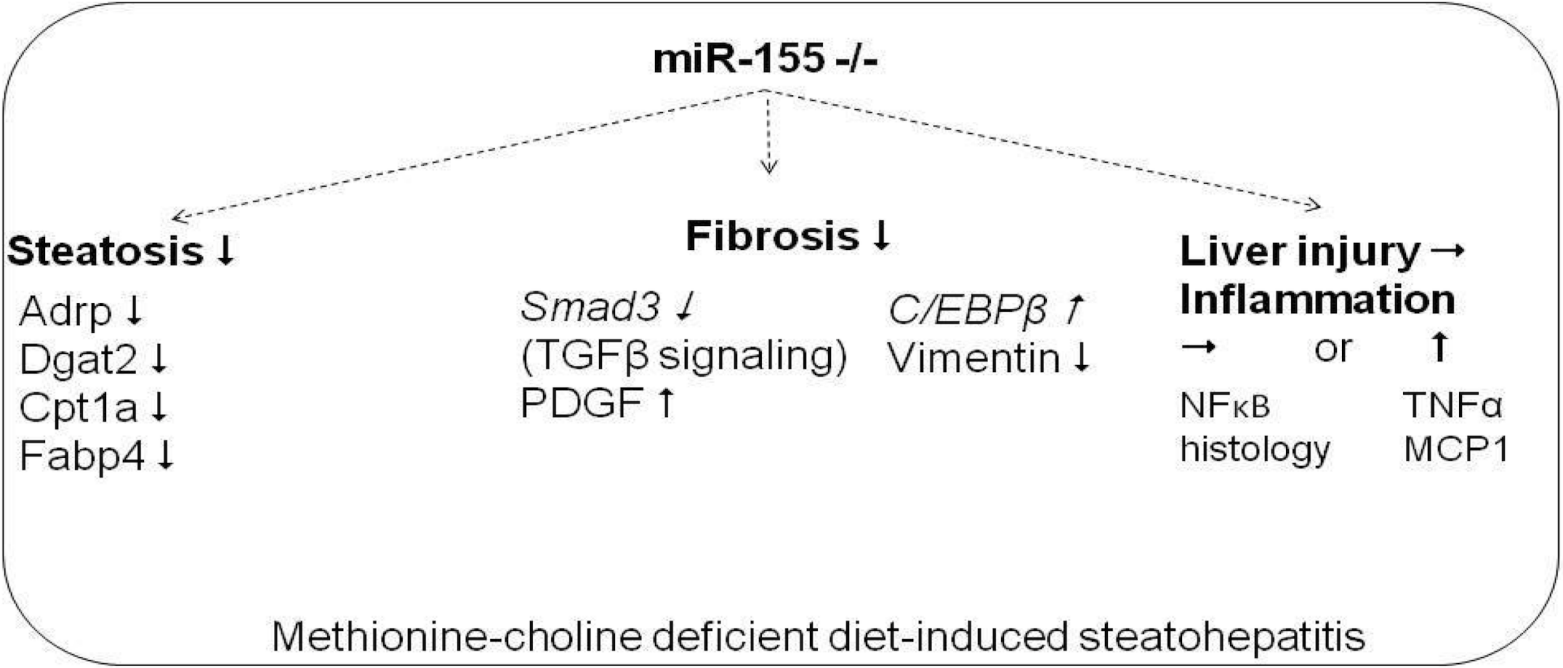
Summary figure: Role of miR-155 in experimental MCD induced steatohepatitis. Putative direct miR-155 targets are in italics.

In conclusion, here we show novel data that miR-155 deficiency attenuates liver steatosis and fibrosis, but not liver injury and inflammation in the MCD model of steatohepatitis (Fig 8). The regulation of steatosis by miR-155 varies depending on the steatohepatitis model; involving genes in TG and VLDL synthesis in methionine-choline deficiency. The regulation of fibrosis is independent of overall liver injury in MCD-steatohepatitis and to a certain extent is detached from inflammation, since miR-155 deficiency did not attenuate hepatic injury, inflammatory cell infiltration or TNFα production. The potential fibrotic mechanisms identified in our model include regulation of apoptosis (caspase-3), Smad3, PDGF and C/EBPβ by miR-155. Finally, our data also underline the importance of the negative regulatory role of miR-155 in the inflammation pathways in NASH.
